# The complete mitochondrial genome sequence of *Trichoderma texanum* (Hypocreales, Sordariomycetes)

**DOI:** 10.1080/23802359.2026.2626067

**Published:** 2026-02-09

**Authors:** Munkhgerel Dalantai, Seung-Yoon Oh

**Affiliations:** a Department of Biology and Microbiology, Changwon National University, Changwon, Republic of Korea; b School of Advanced Biosciences, Changwon National University, Changwon, Republic of Korea

**Keywords:** Mitogenome, endolichenic fungi, fungi, lichen, phylogenetic analysis

## Abstract

*Trichoderma texanum* is a species of saprotrophic and endolichenic fungus in the genus *Trichoderma*. In this study, we sequenced and analyzed the complete mitochondrial genome of *T. texanum* strain CSC21B0438. The mitochondrial genome of *T. texanum* is 31,361 base pairs and comprises 15 protein-coding genes, 27 transfer RNA genes, two ribosomal RNA genes, and one open reading frames. Phylogenetic analysis using the maximum-likelihood method revealed that *T. texanum* is closely related to *T. koningiopsis.* The mitochondrial genome of *T. texanum* reported in this study extends the understanding of its phylogeny and genomic characteristics.

## Introduction

The genus *Trichoderma* Pers. 1794 is an ecologically important saprotrophic fungi that contributes to the degradation of organic matter in soil ecosystems and suppresses soil-borne plant diseases (Harman et al. 2004; Kubicek et al. 2011). Moreover, some *Trichoderma* species enhance plant growth and increase its nutrient uptake efficiency (Yao et al. 2023). Currently, the genus *Trichoderma* is known to have around 469 species among which 382 are currently widely distributed worldwide (Cai et al. 2022; Wang et al. 2024). *Trichoderma texanum* Q.V. Montoya, L.A. Meirelles, P. Chaverri & A. Rodrigues 2016 was first reported from the fungus garden of ant in Texas, U.S.A., and described as a new species based on three molecular markers (ITS, *tef*1 and *rpb*2) together with morphological characteristics (Montoya et al. 2016). *T. texanum* has the distinctive morphological characteristics, including globose to broadly green and smooth conidia and the elongated, relatively large chlamydospores with closely related species (Montoya et al. 2016).

According to a recent study, 47 mitochondrial genomes (mitogenomes) representing 22 species of *Trichoderma* have been reported (Wang et al. 2024). Although previous studies on *T. texanum* have included morphological examinations and nuclear gene-based molecular analyses, studies focusing on its complete mitochondrial genome remain limited (Montoya et al. 2016). In this study, we characterize mitochondrial genome of *T. texanum* strain CSC21B0438 and reconstruct phylogeny of *Trichoderma* based on mitogenome. To date, most mitogenomes of *Trichoderma* have been studied in isolates from soil or plants (Kwak 2020; Özkalekaya et al. 2022; Wang et al. 2024), whereas studies on species isolated from other organisms are scarce. This is the first study to analyze the mitogenome of an endolichenic *Trichoderma* strain, providing a useful resource for understanding the genomic characteristics of *Trichoderma* from diverse environment.

## Materials and methods

**Figure 1.**
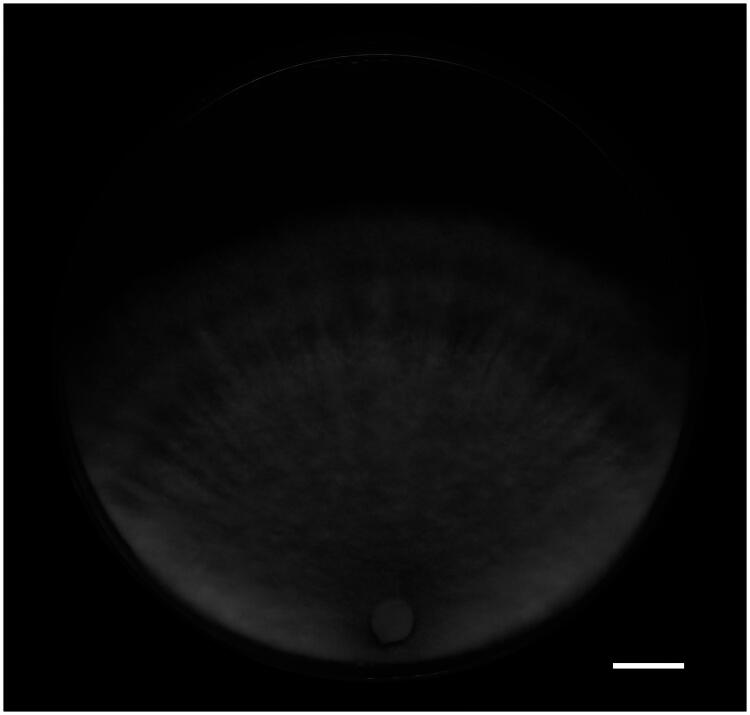
Colony morphology of *Trichoderma texanum* strain CSC21B0438 grown on potato dextrose agar for seven days at 25 °C. Scale bar = 1 cm. Photograph by Yunhyeok Jang.

The studied strain of *T. texanum* was isolated from lichen *Parmotrema* A. Massal. 1860 in Geoje-si, South Korea (34.998783 N, 128.704916 E) and cultured on potato dextrose agar (PDA, Difco Laboratories, Detroit, MI) at 25 °C (Figure 1). The strain CSC21B0438 was identified using the morphological and molecular from the previous study (Oh and Jang 2025) and deposited in the herbarium of Changwon National University (Changwon, South Korea). Genomic DNA was extracted by AccuPrep^®^ Genomic extraction kit (Bioneer, Daejeon, South Korea). The quantity and quality of extracted DNA were measured by Qubit 4.0 (Thermo Fisher Scientific Inc., Waltham, MA) and 2100 Bioanalyzer (Agilent, Santa Clara, CA). The complete mitogenome sequencing was performed using Illumina NovaSeq platform from Macrogen (Seoul, South Korea). The strain CSC21B0438 and its DNA are deposited in the herbarium of Changwon National University, South Korea (Seung-Yoon Oh; syoh@changwon.ac.kr).

Raw reads were quality-filtered and trimmed by FASTP (Chen et al. 2018). The filtered reads were assembled *de novo* using NOVOPlasty v3.6 (Dierckxsens et al. 2017). Using the approach described by Ni et al. (2023), we generated a coverage depth map, which revealed an average sequencing depth of 5440× (Figure S1). MFannot (Lang et al. 2023) and GeSeq (Tillich et al. 2017) were used to annotate mitogenomes. tRNAscan-SE was used to identify transfer RNA (tRNA) genes (Lowe and Chan 2016) and manual curation was performed using Geneious Prime 2021.1 (Dotmatics, Boston, MA). Circular gene map visualized by OGDRAW version 1.3.1 (Greiner et al. 2019). The complete mitogenome sequence of *T. texanum* obtained in this study has been deposited in NCBI (GenBank) under accession number PX395767.

The conserved 14 protein-coding genes (PCGs) were used for phylogenetic analysis (*atp6*, *atp8*, *atp9*, *cob*, *cox1*, *cox2*, *cox3*, *nad1*, *nad2*, *nad3*, *nad4*, *nad4L*, *nad5*, and *nad6*). Amino acid sequences of PCGs were aligned using Clustal Omega (Sievers et al. 2011) and concatenated by using Geneious Prime. The best substitution model for each mitochondrial PCG was determined using ModelTest-NG (Darriba et al. 2020). *Hypomyces aurantius* (Pers.) Fuckel 1870 (KU666552) and *Cladobotryum mycophilum (Oudem.)* W. Gams & Hooz. 1970 (MT108299) were used as outgroup species (Deng et al. 2016; Chen et al. 2020). Phylogenetic analysis was performed using RAxML (Stamatakis 2014) using 1000 bootstrap replicates through the graphical interface raxmlGUI v2.0 (Edler et al. 2021).

## Results

**Figure 2.**
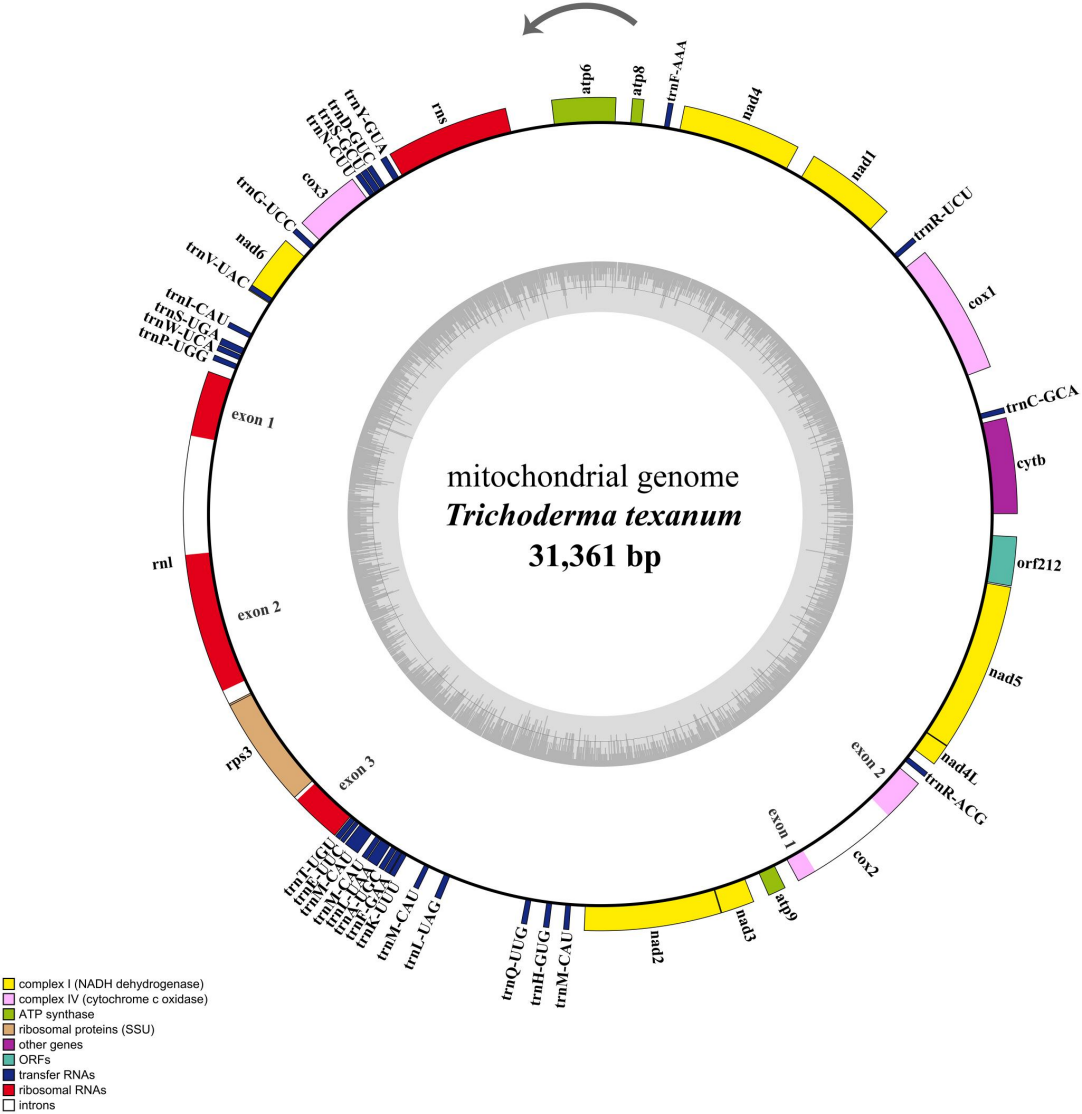
Annotated circular map of the mitochondrial genome of *Trichoderma texanum* strain CSC21B0438. Gene clusters were shown with different color blocks. The complete mitochondrial genome comprised 15 protein coding genes (PCGs), one open reading frame (orf), 27 transfer RNA, and two ribosomal RNA genes. Arrows indicate the transcriptional direction of genes. The inner gray ring depicts the GC content distribution along the mitochondrial genome, illustrating local variation in nucleotide composition.

The complete mitogenome of *T. texanum* strain CSC21B0438 was assembled in a single contig as a closed circular DNA. It was 31,361 bp in length, and overall base composition was A 31.6%, C 12.8%, G 15.5%, and T 35.5%. It comprised 15 PCGs, one open reading frame (orf), 27 tRNA, and two ribosomal RNA genes (Figure 2). Among the PCGs, there are 14 conserved PCGs and one ribosomal protein S3 (*rps3*). There was one PCG (*cox*2) with one intron and large subunit ribosomal RNA (*rnl*) with two introns (Figure S2). The *rps*3 gene was localized within an intron of the *rnl* gene (Figure 2). Our analysis revealed that the *cox1* intron is absent from the mitogenome of *T. texanum*. All of the PCGs were found to have ATG as the predicted initiation codon and TAA as the termination codon, while orf212 was found to start with TTA.

**Figure 3.**
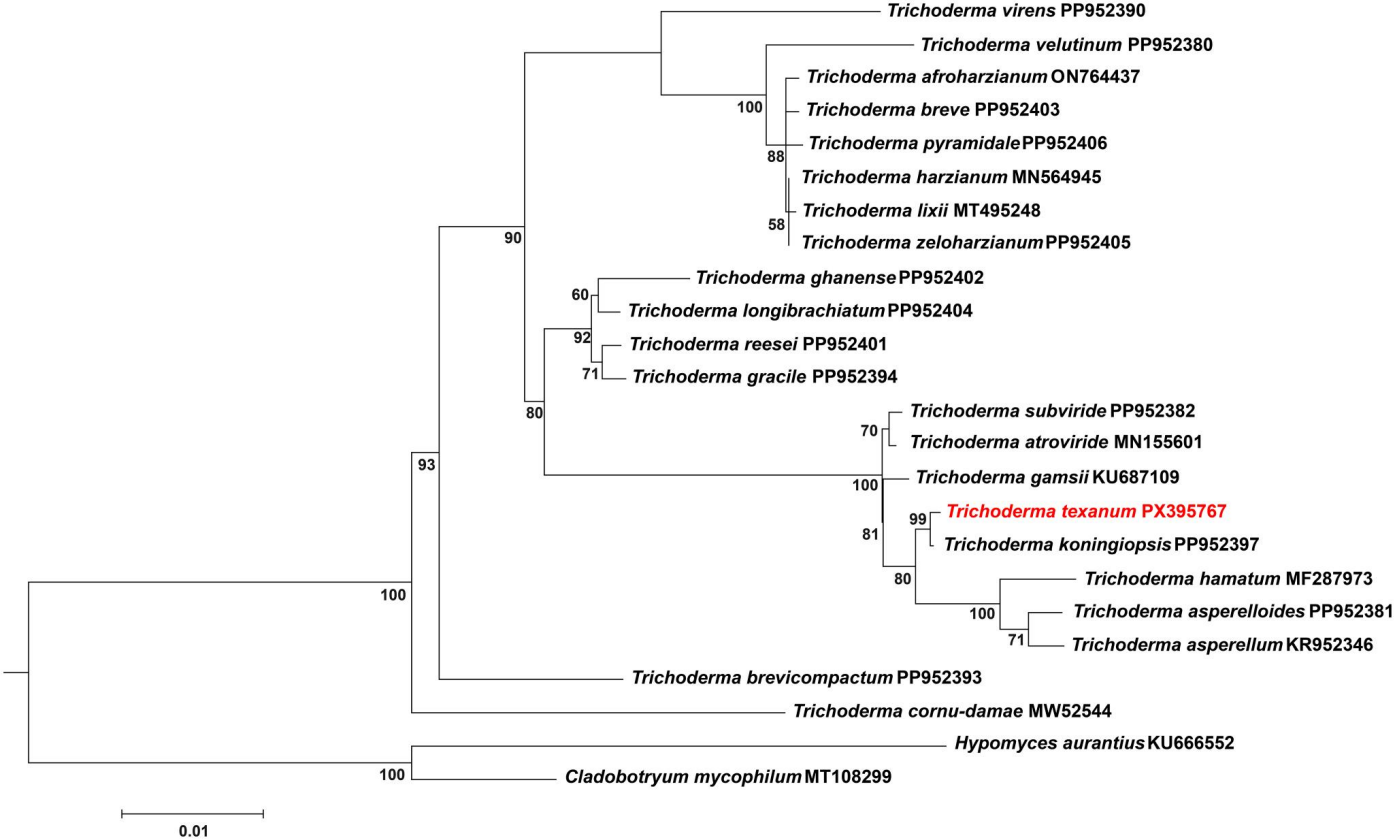
Phylogenetic relationship of *Trichoderma* species based on 14 conserved protein-coding genes (*atp6*, *atp8*, *atp9*, *cob*, *cox1*, *cox2*, *cox3*, *nad1*, *nad2*, *nad3*, *nad4*, *nad4L*, *nad5*, and *nad6*). Numbers below branches indicate maximum-likelihood bootstrap support values. Only bootstrap values >50% are shown. The following mitochondrial genomes were used: *T. koningiopsis*, *T. subviride*, *T. asperelloides*, *T. ghanense*, *T. longibrachiatum*, *T. velutinum*, *T. reesei*, *T. gracile*, *T. virens*, *T. zeloharzianum*, *T. brevicompactum*, *T. pyramidale*, *T. breve* (Wang et al. 2024), *T. asperellum*, *T. hamatum*, *T. atroviride*, *T. gamsii* (Kwak 2020), *T. harzianum* (Kwak 2021), *T. afroharzianum* (Özkalekaya et al. 2022), *T. lixii* (Castrillo et al. 2023), *T. cornu-damae* (Lee et al. 2022), *Cladobotryum mycophilum* (Chen et al. 2020), and *Hypomyces aurantius* (Deng et al. 2016). GenBank accession numbers are provided following the species names.

Phylogenetic analysis was performed on the conserved PCGs of mitogenomes of 22 *Trichoderma* species (Figure 3). For ML analysis, the model of substitution selected was JTT for *cob*, *cox1*, *cox2*, *cox3*, *atp6*, *atp9*, *nad1*, *nad4*, and *nad3*; WAG for *atp8* and *nad2*; CPREV for *nad6* and *nad5*; and BLOSUM62 for *nad4L*. The phylogenetic tree showed the *Trichoderma* genus formed a monophyletic clade. Among the genus, *T. cornu-damae* (Pat.) Z.X. Zhu & W.Y. Zhuang 2014 was early separated, and *T. texanum* is in close relation to *T. koningiopsis* Samuels, Carm. Suárez & H.C. Evans 2006 with a well-supported clade (bootstrap value = 99%).

## Discussion and conclusions

We report, for the first time, the sequencing and characterization of the complete mitogenome of *T. texanum*. Many of the mitochondrial genomic features identified in *T. texanum* were consistent with those reported for other *Trichoderma* species. The mitogenomes of *Trichoderma* species range from 26,276 to 94,608 bp in length (Lee et al. 2022; Wang et al. 2024), and *T. texanum* falls within this range (31,361 bp). In most *Trichoderma* species, the stop codon of *nad4L* (TAA) and the start codon of *nad5* (ATG) overlap by a single nucleotide, with the shared adenine (A) functioning as both the terminal base of *nad4L* and the initial base of *nad*5 (Kwak 2021; Özkalekaya et al. 2022). This single base overlap was also observed in *T. texanum* genome. In fungal mitogenomes, the *rps3* gene is often located within the intron of the *rnl* gene (Özkalekaya et al. 2022; Oh 2024). Consistent with previous reports, this study also detected the *rps3* gene within the intron of the *rnl* gene. In contrast to these shared features, one notable difference was observed in the *cox1* gene. The *cox1* gene contains intron in some *Trichoderma* species (Wang et al. 2024); however, in our studies, the intron of *cox1* gene is not detected in *T. texanum*. In the context of the phylogenetic distribution of this intron, its absence in *T. texanum* suggests a possible lineage-specific intron loss. The results of the phylogenetic analysis confirmed that the genus *Trichoderma* forms a well-supported monophyletic clade and that *T. texanum* is closely related to *T. koningiopsis*, in agreement with previous reports (Montoya et al. 2016; Kullnig-Gradinger et al. 2002). In conclusion, this study expands our knowledge of the complete mitogenome of *T. texanum* and provides a foundation for future research to fully explore its potential applications.

## Supplementary Material

Supplemental Material

Supplemental Material

## Data Availability

The genome sequence data that support the findings of this study are openly available in GenBank of NCBI under the accession number PX395767. The associated BioProject, BioSample, and SRA numbers are PRJNA1329133, SAMN51367437, and SRR35417604, respectively.
