# Supplementary material for: The complete mitochondrial genome sequence of Trichoderma texanum (Hypocreales, Sordariomycetes)
Source: Mitochondrial DNA B Resour. 2026 Feb 9;11(3):383–7. doi: 10.1080/23802359.2026.2626067 (PMC12888358; doi:10.1080/23802359.2026.2626067)
Supplement: Supplemental Material [file TMDN_A_2626067_SM6864.pdf]

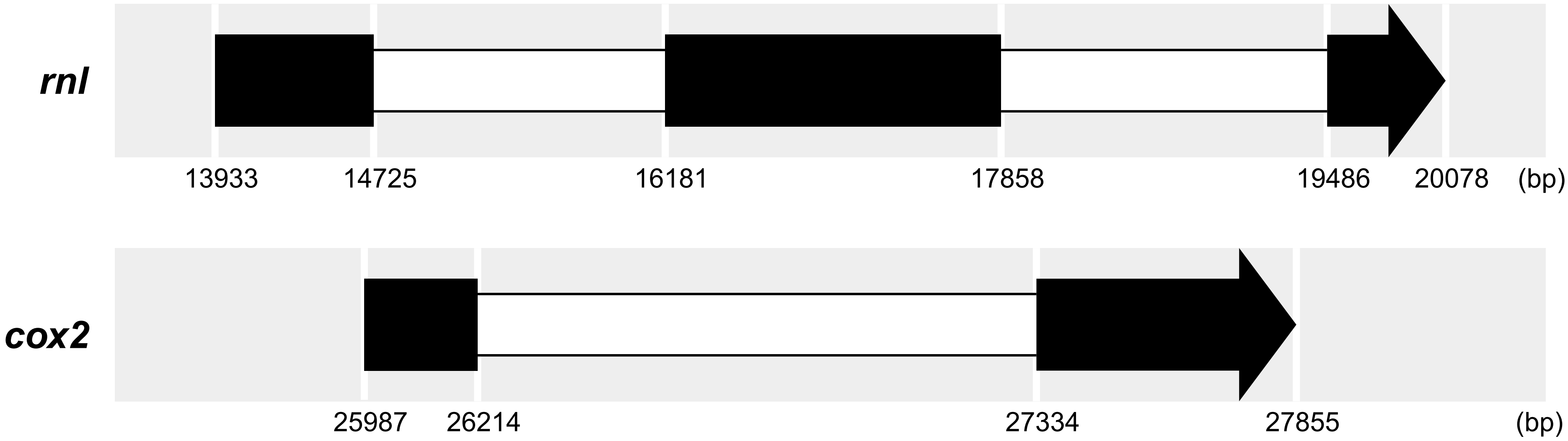

Figure S2. Schematic diagrams illustrating the cis-splicing structures of the mitochondrial rnl and cox2 genes. Exons are depicted as black blocks, whereas introns are shown as white blocks. Numbers displayed below the diagrams denote the genomic positions (bp) corresponding to exon–intron boundaries.
