# Supplementary figures and images for: The complete mitochondrial genome sequence of Trichoderma texanum (Hypocreales, Sordariomycetes)
Source: Mitochondrial DNA B Resour. 2026 Feb 9;11(3):383–7. doi: 10.1080/23802359.2026.2626067 (PMC12888358; doi:10.1080/23802359.2026.2626067)

# Sequencing Depth and Coverage Map

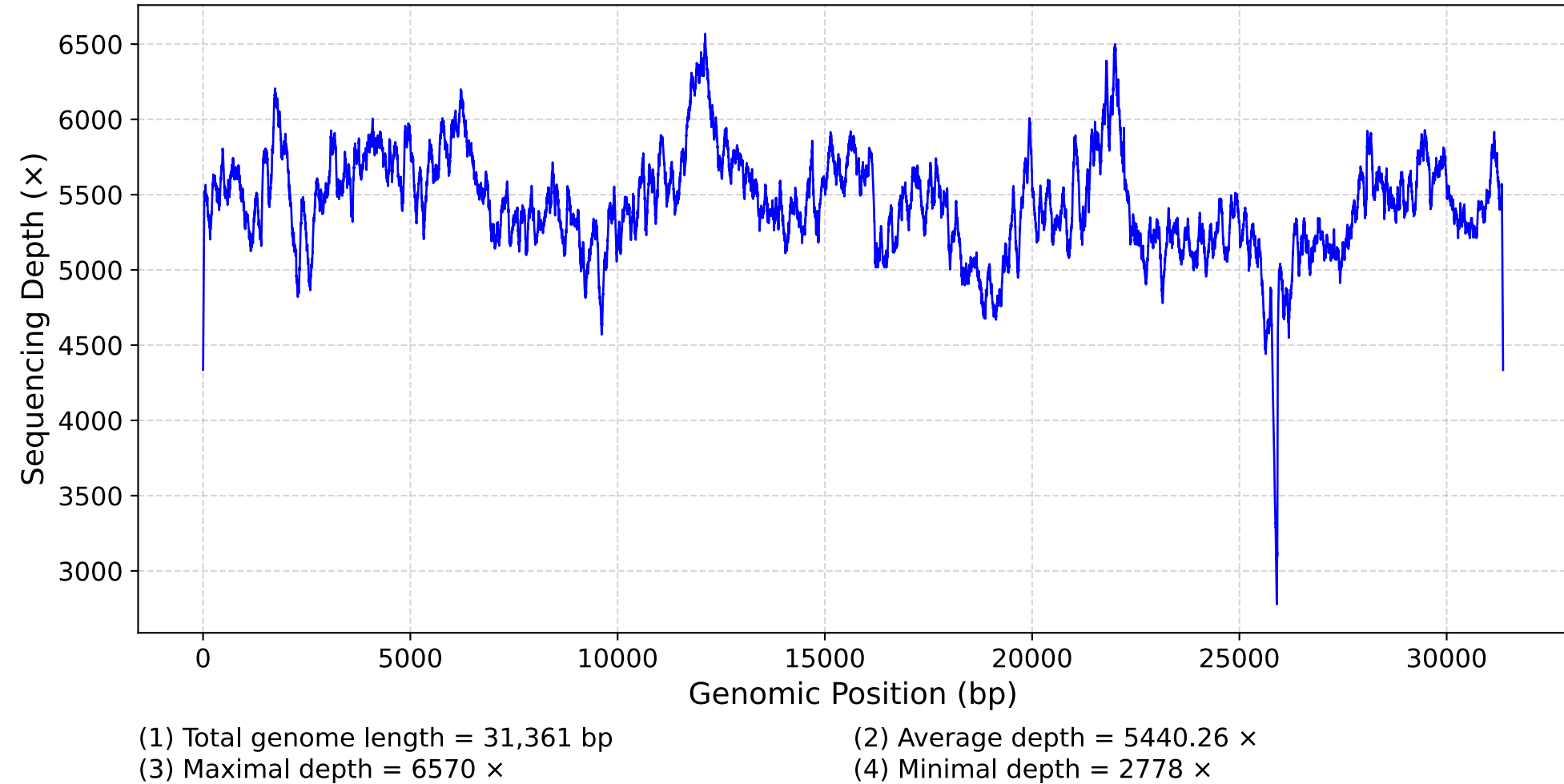

**Figure S1. The sequencing depth and coverage map.**

Supplement: Supplemental Material [file TMDN_A_2626067_SM6863.pdf]
